# An Environmental Friendly Tapioca Starch-Alginate Cultured Scaffold as Biomimetic Muscle Tissue

**DOI:** 10.3390/polym13172882

**Published:** 2021-08-27

**Authors:** Che-Wei Lin, Po-Ting Wu, Kuan-Ting Liu, Yu-Jui Fan, Jiashing Yu

**Affiliations:** 1 School of Biomedical Engineering, Taipei Medical University, Taipei 10675, Taiwan; jericho0511@gmail.com; 2 Department of Chemical and Biomolecular Engineering, University of Pennsylvania, Philadelphia, PA 19104, USA; potingwu@seas.upenn.edu; 3 Department of Chemical Engineering, National Taiwan University, Taipei 10617, Taiwan; b05504018@ntu.edu.tw

**Keywords:** starch, alginate, composite scaffold, myogenic differentiation

## Abstract

Natural porous scaffolds have been studied and developed for decades in biomedical science in order to support cells with a simulated extracellular matrix in natural tissue as an ideal environment. Such three-dimensional scaffolds provide many degrees of freedom to modulate cell activity, such as porosity, pore size, mechanical strength, biodegradability, and biocompatibility. In this study, a porous, three-dimensional material of alginate incorporating tapioca starch was fabricated. A particular freeze-gelation method was applied to homogenously mix starch in the alginate, and the concentration was controllable. This pure natural composite porous scaffold was characterized physically and biologically. The synergistic functions, including biocompatibility, biodegradability, cell adhesion, and cell proliferation, were also investigated. A myogenic differentiation model further verified that the composite porous scaffold provided a suitable environment, supporting the differentiation effect in the myogenic process. The positive results demonstrated that this novel material has the potential to serve as a biomedical or clean meat appliance.

## 1. Introduction

Biomaterials as cell culture scaffold and tissue engineering offer an alternative approach to repair and regenerate damaged human tissue. The technique is a new solution to sufferers from transplants or treatments. Tissue engineering is targeted to be applied to the regeneration of diverse tissues such as the liver, small intestine, muscle, cardiovascular structures, nerve, and cartilage [1,2]. Although tissue engineering is a promising therapy in repair and regeneration areas, the selection and collocation of materials and cells for the corresponding purpose is still a significant issue. An ideal scaffold must provide a frame as a base, which allows cells to attach, proliferate and differentiate to target tissue cells, leading to the repair of organs. However, the features of scaffolds such as surface traits, internal structure, chemical properties, and mechanical properties have a great effect on cellular behavior and organ regeneration [3,4]. Even though the development of tissue engineering is continuing, it already has high competitiveness in the clinical therapy and biomedicine area.

Muscle damage frequently occurs in exercise [5,6], disease [7,8], and trauma [9,10]. Moreover, traumatic injury has been commonly observed in patients in clinical conditions. Common traumatic injuries damage tissues and organs with external forces, causing tear or rupture of the muscle fibers on muscle and tendons. Moreover, blood vessels can also be broken, causing bleeding, bruising, tissue necrosis, and even more, major trauma that has the potential of prolonged disability or death [11,12,13]. The repair of a large area in muscle tissue, especially in the abdomen and with limb defects, is currently a challenge for surgeons and leads to a big problem for patients. For clinical therapies, auto-/allo muscle grafting is the most common therapy in patients. Muscle grafting is to transfer self-tissues from other distant normal muscle sites to injury sites or to receive muscle transplants from donors [14,15,16,17]. These treatment methods are commonly implemented for the surgical repair of muscle-tissue defects, but there are still potential risks in muscle graft transplantation, such as short procedure duration [18,19], high donor site morbidity [20,21], graft rejection reaction [22,23], potential complications [24,25,26], and bacterial or viral infection, etc. [27,28]. Besides using self-tissues as implants in surgery, a novel alternative is to in vitro construct implantable biomaterials with myoblasts to undergo myogenesis directly at the damage site. These are mainly made of natural polymers [29,30,31], synthetic polymers [32,33], or ECM. Refs. [34,35] have attempted to create a microenvironment niche to favorably control the behavior of resident cells. Finally, these biological scaffolds have been implanted in an attempt to improve the repair of muscle tissues in human beings.

Alginate belongs to a biopolymer family. As a polysaccharide, alginate is made up of thousands of units of guluronate and mannuronate, and the ratio of sugars determines the strength of the constructed gel [36,37]. Alginate is a highly hygroscopic, highly biocompatible, and nontoxic material with ease of gelation, so an alginate-based material is known to possess characteristics such as flexibility, tensile strength, compressibility, and resistance to tearing [38,39]. In addition, most composite scaffolds of alginate created via the freeze-drying method demonstrate porous and spongy structures. These meticulous network structure can increase the mechanical strength and stability of the scaffolds [40,41]. Meanwhile, cells can easily move through the holes in scaffolds. Moreover, the addition of adhesive peptides and natural polymers to alginate moieties improves the delivery of nutrients, supporting the cultivation of multiple cell types, such as myoblasts [42,43], stem cells [44,45], and fibroblasts [46,47].

Starch is the major form of carbohydrate storage in plants, and it is also the most common carbohydrate in the diet, widely found in staple foods such as potato, tapioca, corn, rice, and wheat. Starch consists of two different polymers, amylose and amylopectin. The amylose/amylopectin ratio mainly depends upon the plant variety of the starch. Amylose is a polysaccharide made of α-D-glucose units, bonded to each other through α(1→4) glycosidic bonds; amylopectin has chains of α(1→4) linked glucose arranged in a branched structure with α(1→6) branching links, the chemical structural formulae exhibit dendritic structures [48,49]. In addition, the amylose/amylopectin ratio of content has influences on multiple chemical and physical properties, such as granule size of starch, gelatinization, and viscosity. Higher amylose content of starch possesses temperature plasticity and gelatin property, while viscosity increases with amylopectin content after the gelatinization process. This is an important property related to water absorption and swelling for the related material applications [50,51]. The area of starch investigation has been reported to be a source of potential biomedical materials due to starch’s lower cost, abundance in nature, biodegradability, and hydration property. In the meantime, starch provides a similar physical structure with chemical functions equivalent to protein, so that starch and polysaccharide is another option to construct an extracellular matrix (ECM) [52]. Previous research indicates that starch-based scaffolds or hydrogel supported attachment [53,54], proliferation [55], and differentiation [56]. For instance, a collagen-based hydrogel scaffold with oxidized starch proposed by Yongbin Xu had a significant influence on the cell bioactivity of human adipose-derived stem cells (hASCs) and enhanced in vitro vascular differentiation. Modification of hydrogel while retaining the porous and adhesive property improves the proliferation and differentiation of hASCs and expresses a better distribution of cells within the hydrogel [57]. A hierarchical starch-based fibrous scaffold, obtained by the combination of starch–polycaprolactone (SPCL) micro-motifs and polycaprolactone (PCL) nano-motifs, created an alternately integrating electrospun nanofiber mesh of layers of plotted microfibers. The nanofiber meshes acted as a cell entrapment system, increasing the cell attachment and proliferation efficiency of human osteosarcoma-derived cells [58]. Mirab et al. proposed a highly porous scaffold with cellulose nanofibers and HA nanoparticles included in a starch/PVA matrix cross-linked by citric acid. They proved the improved cell adhesion of the nanocomposite scaffold with MG-63 osteoblast cells through SEM and MTT assay [54]. These results showed that the use of starch in biomaterials provides another degree of freedom on adjusting cell adhesion and differentiation behavior.

As stated above, a successful biological material should be highly biocompatible, avirulence, and able to provide a supporting cell growth environment. Aiming for such a material, we developed a tapioca/alginate composite scaffold. Such a natural composite scaffold would be an ideal material for biomaterial applications. In this work, a multi-layer three-dimensional porous structure, mimicking the microenvironment architecture, was manufactured by cross-linking tapioca starch and alginate in CaCl_2_/ethanol solution, followed by a freeze-gelation method, forming an ideal scaffold with high biocompatibility, biodegradability, and water retention. Subsequently, the porosity structure, physical properties, chemical properties, and biocompatibility of the fabricated composite scaffold were investigated in detail. Finally, we demonstrated the in vitro cellular assay, revealing that the tapioca/alginate composite scaffolds are biocompatible and supporting to cell adhesion and differentiation of C2C12 myoblast. We considered the proposed tapioca/alginate composite scaffold to be a promising material with potential to be widely used in tissue engineering or food engineering

## 2. Materials and Methods

### 2.1. Tapioca/Alginate Composite Scaffold Fabrication

The synthesis process of tapioca/alginate composite scaffold was modified from the protocols of Wang et al. [59]. In this study, we established different mixing ratios of solution of tapioca (Sigma Aldrich, St. Louis, MI, USA) and alginate (Sigma Aldrich, St. Louis, MI, USA), as shown in Table 1.

Three mL of tapioca/alginate solution was frozen for 16–24 h at −20 °C in a 3 cm/3 cm/3 cm L/W/H plastic mold. The frozen tapioca/alginate ice cube was placed in precooled 8% (*w*/*v*) CaCl2 ethanol solution at −20 °C for 16–24 h to induce gelation of alginate. The scaffold was then taken out of the solution and washed with phosphate-buffered saline (PBS). Next, the scaffold was removed from the PBS, placed in 70% (*v*/*v*) ethanol solution for further deposition of polymers in the structure at room temperature for 24 h, and washed with PBS to remove the remaining unreacted CaCl_2_. The scaffold was air-dried at room temperature and kept in a drier bin for further use.

### 2.2. Characterization of Tapioca/Alginate Composite Scaffold

The chemical and physical properties of the tapioca/alginate scaffolds were then characterized. To verify the chemical composition, a Nicolet NEXUS 470 FT-IR spectrometer (Thermo, Waltham, MA, USA) equipped with a Single Reflection ATR accessory was used, and the spectral data were collected in the wavenumber region from 650 cm^−1^ to 4000 cm^−1^. The surface morphology and structures of the tapioca/alginates scaffolds were observed by a NovaTM NanoSEM 230 scanning electron microscope (Thermo, Waltham, MA, USA). For scanning electron microscopy (SEM) pretreatment of scaffolds, the scaffolds were freeze-dried for 18–24 h, coated with gold by sputter deposition, and then observed at 300× magnification under an SEM. Images and pore size measurements were then processed and analyzed via Image J software (ImageJ 1.47).

Water absorption experiment was carried out by soaking scaffolds in PBS at room temperature for the studied time durations of 0, 1, 2, 4, 8, 12 and 24 h. After soaking, the scaffolds were then taken out from the solution. Excess PBS was removed with filter paper, and the weights of the wet scaffolds were measured. The water absorption percentage (W%) of these scaffolds was calculated using the following formula:

W% = (W wet − W dry)/W dry × 100%

To assess the degradability, tapioca/alginate composite scaffolds were soaked in PBS for the in vitro biodegradability study. All scaffold groups were incubated with 3 mL PBS solution placed in a 6-well plate at 37 °C. The scaffolds were removed from PBS solution after 0, 1, 2, 4, 8, 12, and 24-day incubation and dried in an oven at 50 °C for 4 h. The weights of the scaffolds (Wt) were measured after drying, and the remaining mass percentage with respect to initial dry weight (W_0_) was calculated as:

Remaining W% = (Wt/W_0_)/W_0_ × 100%

### 2.3. Compressive Mechanical Test

The mechanical properties of the tapioca/alginate composite scaffolds were measured by Elastic Modulus Load Cells (LTS-200GA, Kyowa, New York, NY, USA). Samples were of the shape of 3.0 cm/3.0 cm/0.30 cm L/W/H. The samples were wetted by immersing in water before measurement. Each scaffold was compressed at a rate of 10 mm/min to generate stress–strain curves. Young’s modulus was calculated from the initial linear region of the stress–strain curve. A minimum of three samples was tested for each type of scaffold.

### 2.4. Cell Culture and Seeding

Mouse C2C12 myoblasts (Bioresource Collection and Research Center, BCRC, Taiwan) were cultured in Dulbecco’s Modified Eagle Medium (DMEM/high glucose, Thermo Fisher, Waltham, MA, USA) supplemented with 10% (*v*/*v*) fetal bovine serum (FBS, Thermo Fisher, Waltham, MA, USA), 1% (*v*/*v*) Antibiotic-Antimycotic (Thermo Fisher, Waltham, MA, USA), and 2 mM l-glutamine (Lonza, Basel, Switzerland). The medium was renewed every two to three days. The tapioca/alginate composite scaffolds were assessed for cell compatibility and cell differentiation. All the scaffolds (20 mm in length, 15 mm in width, 5 mm in height) were sterilized with a UV sterilization box (wavelengths between 255 and 280 nm) and then washed with PBS 3 times. Finally, the scaffolds were immersed in differentiation medium containing DMEM/HG medium, 2.5% (*v*/*v*) horse serum (HS, Sigma-Aldrich, St. Louis, MI, USA), 1% (*v*/*v*) Antibiotic-Antimycotic, and 2 mM l-glutamine to promote fusion into myotubes at 25 °C in 6-wells for 10 min before C2C12 (1 × 10^6^/well) were seeded onto the scaffolds. After 6 h incubation at 37 °C, the scaffolds were complemented with 3 mL culture medium. The medium was renewed every two to three days. All the cell experiments were performed in a normal physiological condition at 37 °C and 5% CO_2_.

### 2.5. Cell Viability Assay

The cell viability of C2C12 (1 × 10^6^/well) in tapioca/alginate composite scaffold was assessed using a CCK-8 assay kit (Abcam, Cambridge, MA, USA). After removal of culture medium and PBS wash, we estimated the cell viability in the scaffolds at selected time points (day 1, day 4, day 7, and day 14). An amount of 300 μL of working solution CCK-8 labeling solution was added into the well first, and then the scaffolds were incubated in the incubator for 30 min at 37 °C. The absorbance at a wavelength of 460 nm was measured by an ELISA reader (Hiperion MPR4, Germany).

### 2.6. Cell Viability and Proliferation Visualization of C2C12

To exhibit C2C12 myoblast cell viability and proliferation visualization in tapioca/alginate composite scaffolds, assessment was performed using a MitoBright LT antibody. The C2C12 (1 × 10^6^/well) were stained with MitoBright LT green dyes (1:500, Dojindo, Rockville, MD, USA) for 1 h before seeding on composite scaffolds and we assessed the cell viability and proliferation in the scaffolds at day 5. Finally, the samples were washed 3 times and cell location analyzed by nuclear staining with Hoechst 33342 (1 mg/mL, 5 min at 37 °C). Images were acquired with a Zeiss LSM780 confocal system (Zeiss, Oberkochen, Germany).

### 2.7. Immunofluorescence Staining

The expression levels of myoblasts and myotubes were detected after myogenic differentiation of C2C12 cells. After cells were cultured on tapioca/alginate composite scaffolds for 14 and 21 days, the scaffolds were washed with PBS. The samples were fixed with 4% paraformaldehyde (PHA) for 12–14 h at 4 °C. The cells are penetrated using 1% Triton-X100/PBST (phosphate buffered saline with Tween^®^20) for 5 min and blocked by 5% BSA/PBST (Bovine serum albumin, BSA, Sigma Aldrich, St. Louis, MI, USA) for 2 h. The samples were stained with Myosin Heavy Chain antibody (MHC) (1:100, R&D, Minneapolis, MN, USA), MyoD antibody (1: 200, R&D, Minneapolis, MN, USA), and MyoG antibody (1:200, R&D, Minneapolis, MN, USA) for 12–16 h at 4 °C. F-actin was stained with rhodamine-conjugated phalloidin (1:100, Abcam, Cambridge, MA, USA) at room temperature for 30 min. After rinsing with PBST, the samples were stained with secondary antibodies anti-rabbit/mouse IgG ((green and red)) 1:200, Abcam, Cambridge, MA, USA) at 37 °C for 40 min. The samples were washed 3 times and nuclei were counterstained with Hoechst 33342 (1 mg/mL, 5 min at 37 °C). Images were acquired with a Zeiss LSM780 confocal system (Zeiss, Oberkochen, Germany).

### 2.8. Quantitative PCR (qPCR)

Real-time PCR was used to assess the mRNA expression of myogenic differentiation of C2C12 cells (MHC, MyoD, and MyoG) using SYBR-Green dye (Applied Biosystems, Waltham, MA, USA) for the detection of the DNA. Total RNA was extracted using TRIzol RNA Isolation Reagents (Thermo Fisher, USA). Purified mRNA product was extracted using PureLink RNA Mini Kit (Thermo Fisher, USA). cDNA synthesis was performed with a QuantiTect Reverse Transcription Kit (Qiagen, Germantown, MD, USA). The real-time PCR reaction was performed using a RT2 SYBR^®^ Green qPCR Mastermixes (Qiagen, USA). The sequences of primers can be found in Table 2. The cDNA amplification was conducted by a StepOne™ Real-Time PCR System (Thermo Fisher, Waltham, MA, USA). The assay was replicated in 4 independent experiments.

### 2.9. Statistical Analysis

The data were analyzed via the ANOVA test and by using GraphPad Prism 7 software (GraphPad software, San Diego, CA, USA). Data were presented as the mean ± SD (standard deviation). A value of *p* ≤ 0.05 was considered statistically significant.

## 3. Results and Discussion

### 3.1. Characterization of Tapioca/Alginate Composite Scaffold

**Figure 1:**
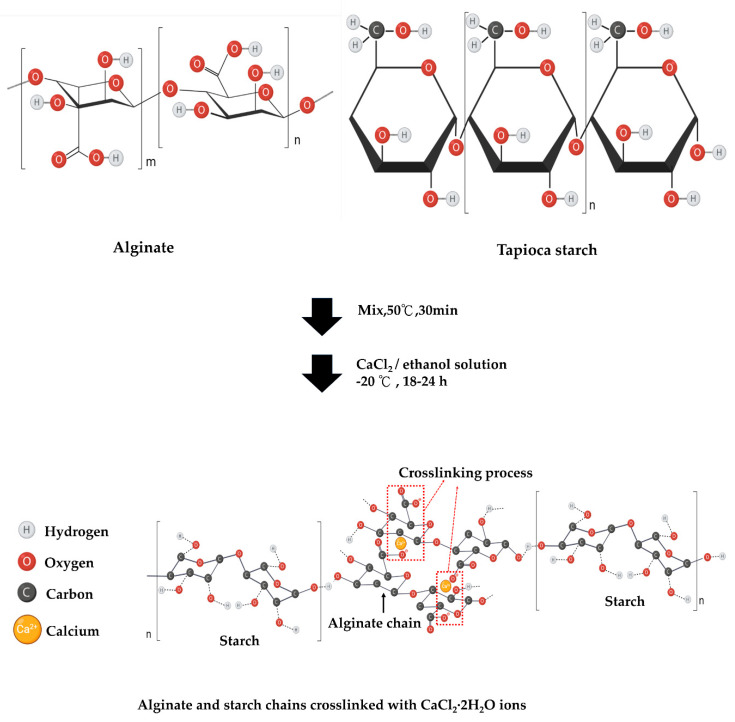
Graphical representation of the chemical interaction of calcium ion and hydrogen bond incorporated tapioca/alginate composite scaffolds.

**Figure 2:**
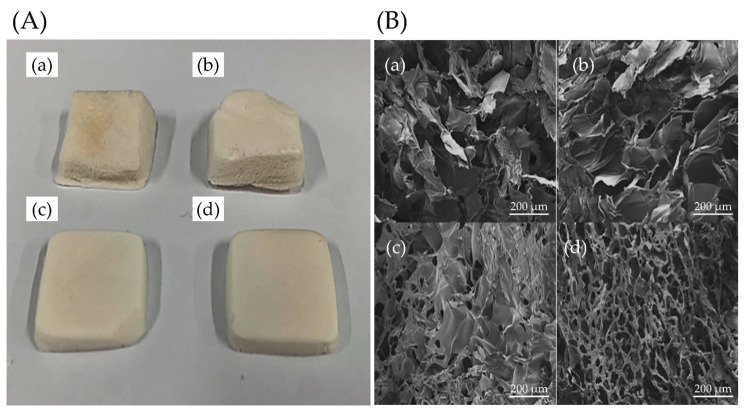
Morphological characterization of the tapioca/alginate composite scaffold. (**A**) Surface morphology of TA scaffolds. (**B**) Cross-sectional view of the TA scaffolds taken by scanning electron microscope. The different concentration ratio of starch of the tapioca/alginate composite was (**a**) TA1, (**b**) TA2, (**c**) TA3, and (**d**) TA4, respectively.

**Figure 3:**
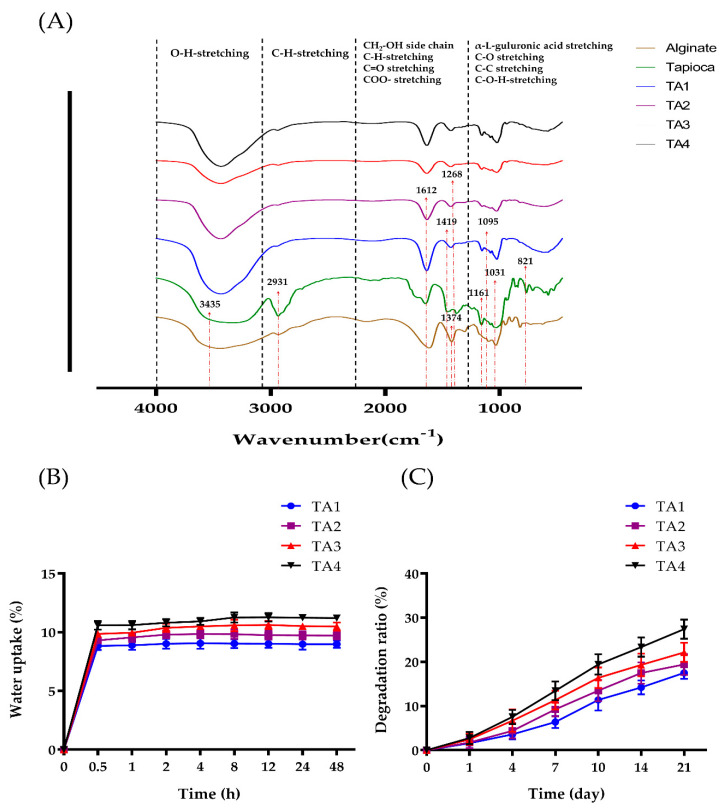
Structural characterization of TA composite scaffolds. (**A**) FTIR spectra of alginate, tapioca, and TA scaffolds. (**B**) Water uptake percentage of dried TA scaffolds. (**C**) In vitro degradation behavior of TA scaffolds in PBS solution.

In this research, a novel, biocompatible material for cell growth promotion was developed. We fabricated tapioca/alginate composite scaffolds (TA scaffolds) using an in-house developed freeze-gelation. Tapioca and alginate powder were dissolved in an aqueous solution at different concentrations and frozen in the mold at −20 °C for 24 h. Frozen samples were soaked by CaCl_2_/ethanol solution for 24 h. This procedure produced a porous type material that could be modified by CaCl_2_/ethanol treatment (Figure 1). By using this method, we obtained four types of composite scaffolds: TA1, TA2, TA3, and TA4 (Table 1). For further analysis, we first observed these scaffolds in the dried states. Four different TA composite scaffolds showing a creamy white and macroscopically uniform exterior were obtained after drying as shown in Figure 2A. The surface of the TA4 group looked rather smooth, without obvious cracks or holes. Compared with the TA4 group, the surfaces of TA1, TA2, and TA3 groups were rough, with some naked-eye visible pleated structure. The morphology of the tapioca/alginate scaffolds was analyzed by SEM at 250× magnifications, and all TA scaffolds presented porous structures, showing that the scaffolds had great structural consistency (Figure 2B). The pore size analysis by Image-J software showed that the pore sizes of the TA1, TA2, TA3, and TA4 scaffolds were in the range of 103 ± 19 μm, 108 ± 15 μm, 118 ± 22 μm, and 124 ± 19 μm, respectively. Relevant previous studies reported that using composite scaffolds with pore sizes of 85–190 μm is suitable for cell attachment [60]. As shown in Table 3, all the fabricated alginate scaffolds with different concentrations had suitable pore size structures for cell attachment. Nevertheless, compared with other groups, the morphology of the TA4 groups had a more uniform porous structure with interconnected pore networks (Figure 2B), which could be attributed to the higher concentration of tapioca. Generally, increasing the viscosity by raising the concentration of starch may promote gelation and further increase the strength of the structure, with increasing pore size [55]. Ideally, TA4 group should be able to facilitate several favorable properties such as cell adhesion and nutrient or oxygen diffusion. FTIR spectra were used to determine the interactions between tapioca and alginate in composite scaffolds (Figure 3A). All groups exhibited strong characteristic peaks of O–H stretching at 3435 cm^−1^. The presence of the O–H band confirmed the chemical structure of the tapioca starch and alginate in the composite scaffolds, indicating that the ability to form hydrogen bonds was preserved, proved by two signals appearing at 1612 cm^−1^ and 1268 cm^−1^; the first peak was due to the C=O stretching vibration, and the second one was due to CH_2_–OH side chains. The carbonyl signal (C=O) can possibly be attributed to carboxylic acid, ketone, or aldehyde groups. The tapioca starch scaffold presented a peak for CH_2_–OH side chains at 1268 cm^−1^, and the C=O stretching vibration of the amide group was found at about 1612–1660 cm^−1^, while the 1410–1485cm^−1^ peak was attributed to a C–H group. The peaks at 1161 cm^–1^ and 1095 cm^–1^, observed in the spectra of all the tapioca starch scaffolds, correspond to C–C groups and C–O stretching vibrations. A small peak at 1419 cm^−1^ and another peak at 1612 cm^−1^ were identified and attributed to the symmetric and asymmetric stretching of the carboxylate group −COO−, respectively. The presence of these two bands suggested the involvement of COO− groups in the Ca^2+^ mediated process of alginate reticulation; the peak at 1031 cm^−1^ was assigned to the C–O–H group. The characteristic peak of α-L-guluronic acid and β-D-mannuronic acid group at 821 cm^−1^ was the signature of alginate. In previous studies of starches, Awolu et al. found several functional groups presented in amylose and amylopectin such as the O–H group, the C=O aldehyde, and C–H alkanes, where the results proved that the structure of starch in these composite scaffolds remained after crosslinking procedure [61].

The porosity of TA scaffolds is of significance for efficient cellular behavior. Low porosity may limit cell adhesion and migration in biodegradable materials [62]. To assess the porosity of the TA scaffolds, water immersion porosimetry (WIP) was applied. As shown in Table 3, the porosity of the composite scaffolds exhibited a rising tendency along with the increase of tapioca concentration. The porosity was 52.18 ± 3.68% for TA1, 53.58 ± 4.30% for TA2, 58.93 ± 3.83% for TA3, and 64.65 ± 4.74% for TA4, respectively. Results of the WIP assay showed that the porosity level had an upward trend with increase of tapioca concentration in the TA scaffolds, although there was no significant difference in porosity between the four scaffold experimental groups.

Figure 3B showed the amount of water absorbed by the TA scaffolds with four different tapioca contents. All of the scaffolds quickly absorbed water in the first 30 min, and then reached a plateau after around 2 h of immersion. The water uptake ratio of TA1 and TA2 was found to be 8.98 ± 0.32% and 9.73 ± 0.35%, TA3 and TA4 were found to be 10.48 ± 0.35% and 11.21 ± 0.30 after 48 h. Due to the high water retention and water absorption capacity of alginate, it is frequently used as a natural polymer material [63,64]. Meanwhile, even in the starch family, tapioca still has a high water absorption capacity [65]. The results suggested that the water absorption of TA scaffolds increased with an increase in starch concentration. It is known that a higher degree of cross-linking within the composite scaffold could prevent the uptake of water. With the same amount of functional groups (−OH group), more starch groups involved in the cross-linking process with fewer functional groups available for binding water [66]. However, the increase of starch could enhance the water uptake ability of scaffolds. As a result, the TA4 scaffold showed the highest water uptake ability. Hence, an increase in starch concentration of composite scaffolds led to slightly reduced water absorption. Previous studies showed that starch-based scaffolds can be biodegradable at an appropriate rate to match the speed of cell adhesion and differentiation, so the degradation rate of TA scaffolds under a bio-mimicked environment was examined [62,67]. Four types of TA scaffolds were immersed in PBS solution, and the dried weight was measured at different time points. As shown in Figure 3C, all groups were gradually degraded through time, and the degradation rate of TA4 was higher than TA1. The final degradation rates were 17.53 ± 1.41% for TA1, 19.43 ± 2.41% for TA2, 22.13 ± 2.18% for TA3, and 27.41 ± 2.15% for TA4 groups, respectively, after immersion for 21 days in PBS. Although the degradability of the four scaffolds showed no significant difference, 15–30% degradation in 21 days is an appropriate rate for implants. It showed that the degradation rate of composite scaffolds increased as the tapioca concentration increased. This is likely due to the increased hydrogen bonds and ionic interaction between tapioca starch and water, destroying the crosslinks of the tapioca/alginate network. While the TA4 scaffold contained the most tapioca among the four groups, it had the highest degradability. Nonetheless, the TA scaffolds still provided adequate time for cell adhesion or differentiation.

Cell signaling cascades of cell interactions with biomaterials are significantly influenced by the mechanical properties of biomaterials, determining the growth and behavior of cells, so we assessed the strength of scaffolds by Young’s modulus. The compressive strength was measured by a universal testing machine, and the results are shown in Table 3. The compressive strengths were 509.45 ± 2.22, 531.03 ± 12.42, 574.78 ± 9.80, and 1328.40 ± 22.78 kPa for TA1, TA2, TA3, and TA4, respectively. The compressive strength of the TA4 scaffold was about two times higher than that of the other TA scaffolds. In previous studies, Bose et al. showed that the scaffolds fabricated from amylose-rich starch had stronger mechanical strength. They developed 3D printed starch-HA composite scaffolds with different types of starch, and the scaffold with corn starch, an amylose-rich starch, expressed higher compressive strength. Also, scaffolds with more starch content showed stronger mechanical strength [68]. This confirmed our results on the compressive strength test. Overall, the high concentration of tapioca starch enhanced the compressive strength of the composite scaffold.

### 3.2. Cellular Viability in Tapioca/Alginate Composite Scaffolds

**Figure 4:**
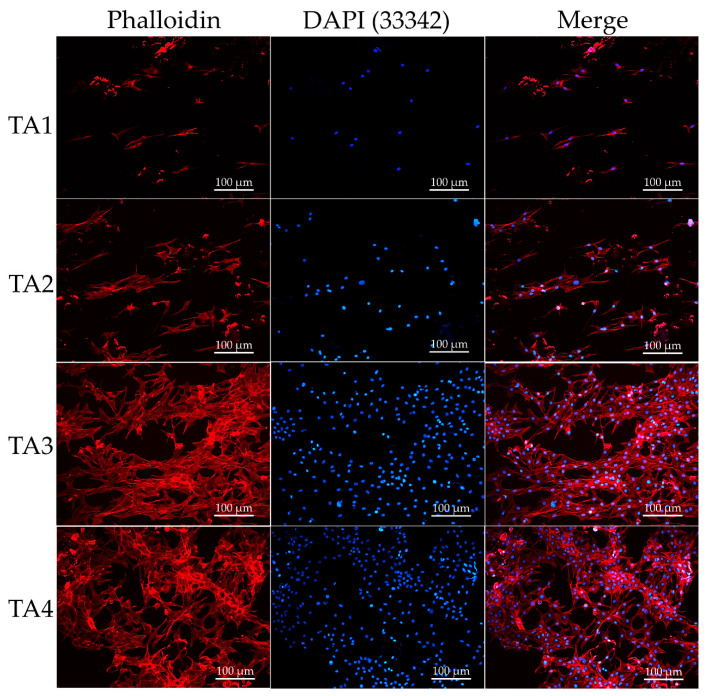
Nuclei and phalloidin staining of C2C12 cells after 5 days in different tapioca/alginate composite scaffolds.

**Figure 5:**
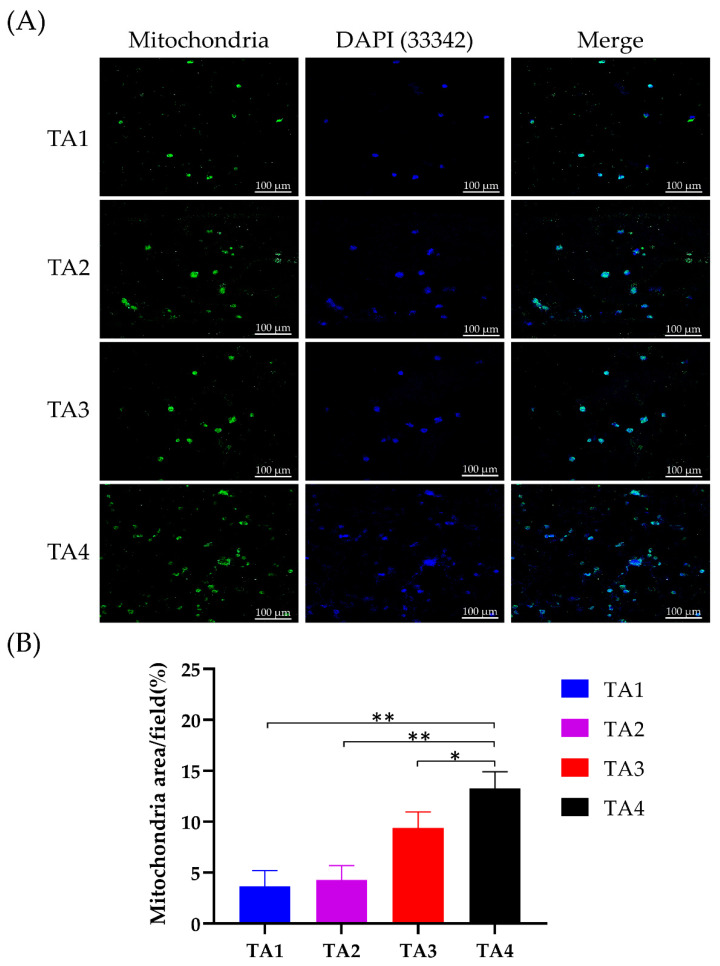
Mitochondrial expression in Tapioca/alginate composite scaffold. (**A**) Immunofluorescence staining to determine mitochondrial expression of C2C12 myoblasts in TA scaffolds at day 5. Hoechst 33342 was used for staining the nuclei. Scale bars represent 100 µm on images (**B**). (**A**) Quantification of immunofluorescent intensity of C2C12 mitochondrial expression in TA scaffolds. Results are represented in means ± SEM. *: *p* < 0.05, **: *p* < 0.01.

The surface properties of composite scaffolds were shown to notably influence the adhesion, proliferation, and viability of cells, and eventually determine the biomedical application feasibility of the scaffolds [55]. To characterize the consequences of cell adhesion and the spreading of C2C12 cells on TA scaffolds, cells were stained by rhodamine-labeled phalloidin and the morphological phenotype of C2C12 cells analyzed as they cultured in the TA scaffolds for 5 days. On the TA3 and TA4 groups, extensive and curtained cytoskeleton was observed in the C2C12 cells, and the cellular network development could be observed in the composite scaffolds (Figure 4). Meanwhile, both the spreading rate and the number of C2C12 were significantly higher than for the TA1and TA2 groups. For the TA1 and TA2 groups, a significant decrease in cell number and spreading ratio was observed after day 5. This result suggests that a high concentration of tapioca starch may promote cell adhesion in TA scaffolds. To investigate the cellular growth of the C2C12 cells across the composite scaffolds, MitoBright LT (Dijindo, Rockville, MD, USA)dyes were used, and the cells were observed by confocal microscopy. MitoBright LT dyes were designed to exhibit mitochondria retention in live cells for long-term localization. Figure 5 shows the mitochondrial expression of C2C12 cells on different TA scaffolds after 5 days with immunofluorescence staining images at 20× magnifications. The staining images showed a green-fluorescent mitochondrial expression in C2C12 cells inside the pore channels and their spread on the TA3 and TA4 scaffolds; the mitochondrial expression of the TA4 group was observably stronger than those of other groups.

**Figure 6:**
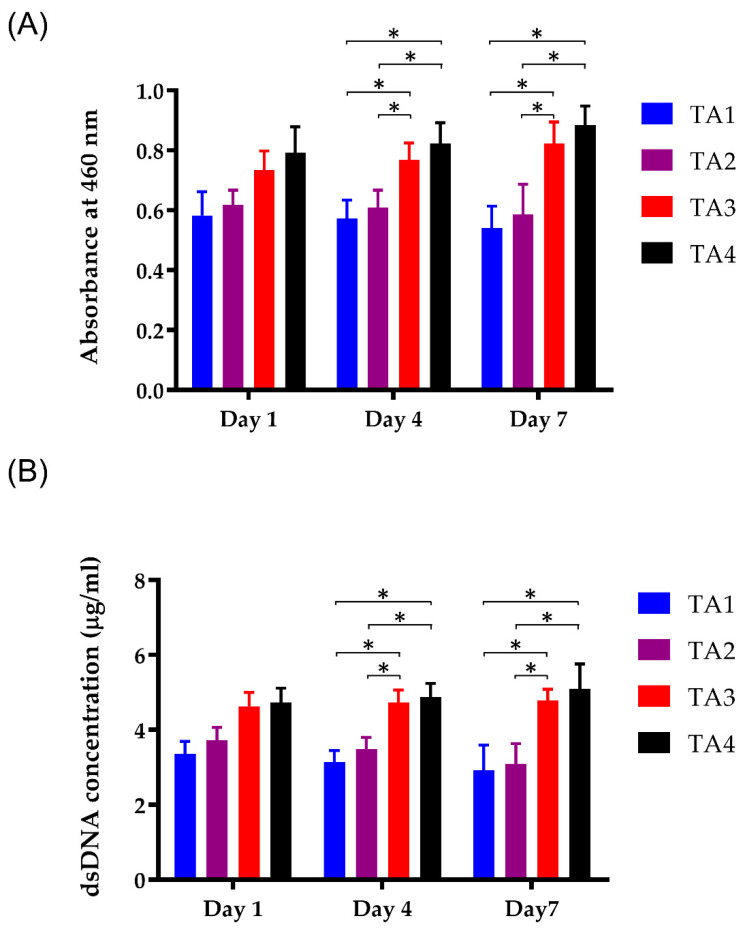
Cell viability of C2C12 myoblasts in contact with the TA scaffolds extraction obtained by (**A**) CCK-8 assay and (**B**) dsDNA assay after different extraction times, including 1-, 4-, and 7-day. *: *p* < 0.05.

On TA1 and TA2 scaffolds, although the mitochondrial expressions within the cells were observed, most of the cells were located only on the surface of the scaffolds and did not show a spread distribution of cells through the entire section of the scaffolds. For the morphology of the fluorescence images, the C2C12 cells in TA scaffolds were regularly distributed (Figure 5A), whereas mitochondria were expressed in the cytoplasm of the C2C12 cells. The intensity of the mitochondrial immunofluorescent signal was quantified (Figure 5B). Compared to other groups, the mitochondrial density of the TA4 group was significantly higher, and there was no significant difference in mitochondria levels between the TA1 group and TA2 group. This is probably due to the larger pore size and lower pore number of the TA4 scaffold. As a result, the larger pore size and lower pore number could enhance the adhesion and proliferation of cells on the TA4 scaffold via better nutrient transport while remaining as the adhesive property to the cells. CCK-8 assay was used to determine the cell viability of C2C12 myoblasts adhering on the different scaffolds (Figure 6A). The cellular viability of C2C12 myoblasts in TA scaffolds increased as the tapioca ratio. The cell survival level of TA3 and TA4 groups was more than 90% on day 4 and day 7, while the decrement in the cell viability of TA1 to TA2 groups can be attributed to the presence of the starch content ratio which decreased apparently. This showed that the fabricated scaffolds with a higher ratio of tapioca had better cytocompatibility with the C2C12 cells. In fact, the starch polymer affected the viability via adhesion of the cells by reducing the tapioca ratio in the scaffolds [53]. Moreover, the data also demonstrated that the utilization of CaCl_2_ as the crosslinking agent showed no significant toxicity on the scaffolds.

Meanwhile, dsDNA assay was applied to detect the changes in the number of cells in the scaffolds (Figure 6B). After 7-day cultivation, the dsDNA content of the TA4 group (5.10 ± 0.67 µg) cultured with C2C12 cells was significantly higher than that of the TA3 group (4.79 ± 0.30 µg), the TA2 group (3.10 ± 0.54 µg), and the TA1 group (2.93 ± 0.67 µg). In contrast to the TA4 group, the dsDNA content of the TA1 and TA2 groups gradually decreased throughout the culture period (TA1: 3.37 ± 0.33µg at day 1, 3.15 ± 0.30 µg at day 4; TA2: 3.72 ± 0.35 µg at day 1; 3.50 ± 0.31 µg at day 4). In addition, the lack of tapioca in the TA1 and TA2 groups resulted in deplorable cell attachment ability, demonstrated by the dearth of fluorescence cell numbers in the composite scaffold at day 5 (Figure 4 and Figure 5). Therefore, the analysis of immunofluorescence staining, CCK-8 assay, and dsDNA assay revealed apparently higher cell viability in the TA4 group throughout the culture period.

### 3.3. Cell Differentiation in Tapioca/Alginate Composite Scaffold

**Figure 7:**
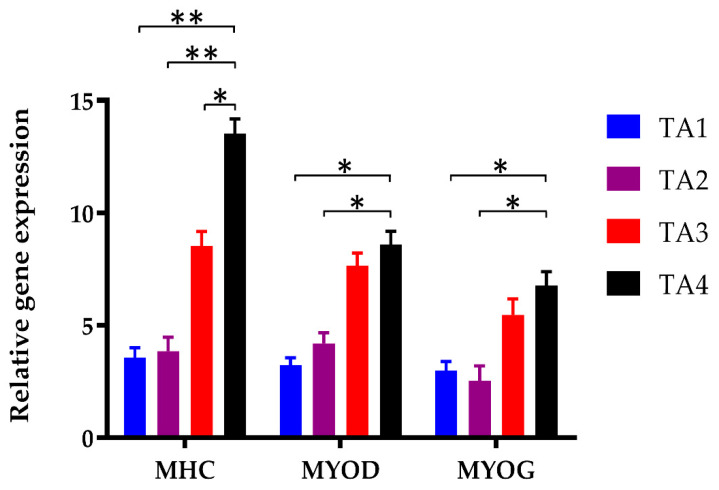
Gene expression profiles of myogenic differentiation associated marker genes in C2C12 myoblasts in TA scaffolds. Graphs represent the relative myogenic gene expression of MHC, Myo D, and Myo G in TA scaffold after day 7. Results were represented in means ± SEM. *: *p* < 0.05, **: *p* < 0.01.

To assess the ability of the tapioca/alginate composite scaffolds to support cell differentiation, we examined the differentiation potential ability of C2C12 myoblasts within the TA scaffolds. To better understand the differentiation of C2C12 during myogenic differentiation in TA scaffolds, we quantified the level of myogenic gene expression of myoblasts in TA scaffolds. Figure 7 shows the level of gene expression of Myosin Heavy Chain (MHC), MyoD, and MyoG in C2C12 after 7-day differentiation. MHC is a skeletal muscle contractile protein that is expressed during muscle development, and is considered to be a marker of the differentiated state, especially in the late differentiation of myoblasts. Meanwhile, MHC expression was also related to multinucleated myotubes formation at the C2C12 myoblast differentiation stage [69]. As the results showed, MHC was expressed in all TA scaffold groups at day 7, while the MHC expression of the TA4 group was significantly more frequent. On the other hand, TA1 and TA2 showed relatively low expression in MHC.

MyoD is a transcription factor of the myogenic basic helix-loop-helix (bHLH) family required for the process of myogenic differentiation. MyoD has also played an important role in skeletal muscle terminal differentiation in myoblast populations [70]. Previous studies have proved that inducing the myogenic differentiation of C2C12 cells was associated with up-regulation of the MyoD gene [71].

MyoG belongs to a muscle-specific helix-loop-helix transcription factor, and it is recognized to be a marker for the entry of myoblasts into the differentiation pathway. Myoblasts with MyoG expression were identified as being at an early stage of myogenic differentiation [72].

Figure 7 shows that MyoD gene expression increased significantly in the TA4 group on day 7; whereas those of TA1 and TA2 groups were extremely low over the same time. The result of MyoG mRNA expression showed that the TA1 and TA2 groups had low levels of MyoG gene expression on day 7. The MyoG gene level of the TA4 group slightly increased, but it was not to any extent of significant difference amongst all the TA groups. Based on this in vitro experiment of myogenic differentiation, the difference between the TA4 group and other groups in terms of mRNA transcript levels of the activation marker MHC, MyoD, and MyoG observable, suggested that the developmental myogenesis process did depend on good growth conditions. Additionally, the high starch concentration of TA scaffolds could offer a favorable cultivation environment for myogenic differentiation.

**Figure 8:**
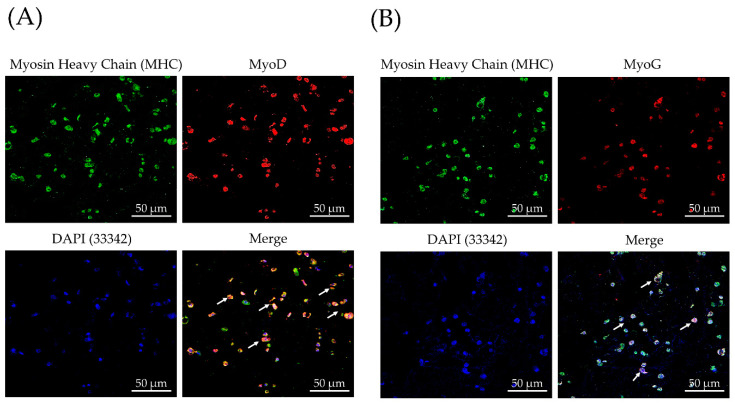
Immunofluorescence staining of myogenic differentiation of C2C12 myoblasts in TA4 scaffolds. C2C12 myoblasts were cultured for 7 days in differentiation medium, and MHC, (**A**) MyoD, and (**B**) MyoG were detected by using the mouse anti-specific mAb. Hoechst 33342 was used for nuclei staining.

To evaluate the cell differentiation of TA scaffolds, we observed the expression of myosin and the presence of fused myotubes in C2C12 myoblast as markers of myogenic differentiation. From the previous result of mRNA gene expression by real-time PCR, we adopted the TA4 group for immunofluorescence staining. A monoclonal antibody (mAb) was used to determine the myogenic differentiation expression of C2C12 cells in TA4 scaffolds, including MHC, MyoD, and MyoG, which have been widely used to mark differentiated myogenic progenitor cells. After 7-day myogenic differentiation, most C2C12 cells expressed an MHC signal (Figure 8).

Furthermore, we observed MHC immunofluorescence was co-localized to a subpopulation of MyoD+ and MyoG+ cells (arrows), respectively (Figure 8A,B). Inspection of intracellular localization indicates that these transcript factors were predominantly located in the cytoplasm at this differentiation stage. It was expected that all three transcription factors would all express. Interestingly, after 7 days with the myogenesis process, the MHC immunofluorescence signal was the most strongly expressed in the C2C12 cell, while MyoD and MyoG were both observable but with a weaker fluorescence signal. The immunofluorescence level was consistent with the result of real-time PC, showing that the presence of MHC expression strongly demonstrated the myogenesis of C2C12 cells on the TA4 group at day 7. Morphologically, all the C2C12 cells and myotubes were of a round shape in the TA4 scaffold. Previous studies have shown that cells adapt their morphology according to the internal and external structure of porous biomaterials, and the pore properties of biomaterials determines how well the cells adhere to scaffolds, which further affects their morphology [54,73]. Consequently, in vitro study of immunofluorescence staining demonstrated that cells are largely circular-shaped, which could be proof that cells tend to bridge small distances between pore walls and pores. Conclusively, cell immunofluorescence staining and real-time PCR confirmed C2C12 myogenic differentiation on the TA scaffold.

## 4. Conclusions

TA scaffolds with different pore sizes were successfully fabricated using freeze-extraction and freeze-gelation methods. Compared with the common freeze-drying method, these methods which we present are more convenient and simpler with no toxic residue remaining in the TA scaffolds. Meanwhile, the TA scaffolds had sufficient water absorption capacity and biodegradability. In addition, the use of TA scaffolds provided higher interconnected porosity, and irregular pore geometry and these factors provide extra degrees of freedom to influence the cellular behavior of the C2C12 cells. As discussed above, the applicability to the cell culture of the prepared scaffolds was also tested. We demonstrated that the TA scaffolds provided a supportive environment for C2C12 cells to adhere and further promote cell viability. Besides, the TA scaffolds resulted in boosted potential cell differentiation of C2C12. Overall, the TA scaffolds offered an optimum platform for 3D cell culture and differentiation. We indicated that the TA scaffolds prepared by the proposed methods had potential applicability for tissue engineering. In addition, as clean meat becomes a popular research field, plant-based cellular agriculture is set to be the future trend of the food industry. TA scaffolds could offer a proper and efficient platform for meat grown in the laboratory.

## Figures and Tables

**Table 1:** Different formulations of tapioca/alginate composite scaffolds.

Tapioca/Alginate (TA)	Tapioca (mg/mL)	Alginate (mg/mL)
0.5:1 (TA1)	10.0	20.0
0.625:1 (TA2)	12.5	20.0
0.75:1 (TA3)	15.0	20.0
1:1 (TA4)	20.0	20.0

**Table 2:** Specific primers for real-time PCR.

Gene	Sequences (5′–3′)
MHC	Forward: 5′-TGTCAA TGCTGCTTTACCTCAA-3′ Reverse: 5′-GCATATCCTGAA AGGCACGAT-3′
MyoD	Forward: 5′-CGGGACATAGACTTGACA GGC-3′ Reverse: 5′-TCGAAACACGGGTCATCATAGA-3′
MyoG	Forward: 5′-CAATGCACTGGAGTTCGGT-3′ Reverse: 5′-CTGGGAAGGCAACAGACAT-3′
Gapdh	Forward: 5′-TGCACCACCAACTGCTTA-3′ Reverse: 5′-GATGCAGGGATGATGTTC-3′

**Table 3:** Physical properties of TA scaffolds.

Tapioca:Alginate (TA)	0.5:1 (TA1)	0.625:1 (TA2)	0.75:1 (TA3)	1:1 (TA4)
Pore size (µm)	103 ± 19	108 ± 15	118 ± 22	124 ± 19
Porosity (%)	58.93 ± 3.83	56.77 ± 2.98	53.59 ± 8.23	52.18 ± 7.62
Young’s modulus (kPa)	38.46 ± 1.27	42.25 ± 6.42	51.78 ± 3.59	68.42 ± 2.18

## Data Availability

Not applicable.
